# Chemical screening identifies the anticancer properties of *Polyporous parvovarius*

**DOI:** 10.7150/jca.78302

**Published:** 2023-01-01

**Authors:** Yun Haeng Lee, Minkyeong Kim, Hyon Jin Park, Ji Yun Park, Eun Seon Song, Haneur Lee, Gahyun Ko, Soonkil Ahn, Hyung Wook Kwon, Youngjoo Byun, Changmu Kim, Jaehyuk Choi, Joon Tae Park

**Affiliations:** 1 Division of Life Sciences, College of Life Sciences and Bioengineering, Incheon National University, Incheon, Korea.; 2 Convergence Research Center for Insect Vectors, Incheon National University, Incheon 22012, Korea.; 3 College of Pharmacy, Korea University, Sejong 30019, Republic of Korea.; 4 Microorganism Resources Division, National Institute of Biological Resources, Incheon 22689, Korea.

**Keywords:** *Polyporus parvovarius*, mycelium culture extract, Protocatechualdehyde

## Abstract

One of the biggest obstacles in cancer treatment is the development of chemoresistance. To overcome this, attempts have been made to screen novel anticancer substances derived from natural products. The purpose of this study is to find new anticancer candidates in the mycelium culture extract of mushrooms belonging to *Polyporus*. Here, we used a high-throughput screening to find agents capable of inhibiting cancer cell proliferation. The culture extract of *Polyporus Parvovarius* mycelium in DY medium (pp-DY) was effective. pp-DY inhibited cancer cell proliferation by inducing apoptosis and S-phase arrest. The anticancer property of pp-DY was not only effective against one type of cancer, but also against another type of cancer. Compound fractionation was performed, and the active ingredient exhibiting anticancer effects in pp-DY was identified as 3,4-dihydroxybenzaldehyde (Protocatechualdehyde, PCA). PCA, like pp-DY, inhibited the proliferation of cancer cells by inducing apoptosis and S-phase arrest. Furthermore, unlike conventional anticancer drugs, PCA did not increase the proportion of the side population that plays the most important role in the development of chemoresistance. Taken together, our data revealed the novel mycelium culture extract that exhibited anticancer property, and identified active ingredients that did not activate a proportion of the side population. These novel findings may have clinical applications in the treatment of cancer, particularly chemo-resistant cancer.

## Introduction

Mushrooms are not only used for edible purposes, but are also considered as functional foods with health benefits 1. Mushrooms produce primary metabolites for growth and reproduction, including the production of nucleic acids, proteins, and energy. Mushrooms also produce secondary metabolites, low molecular weight compounds, that have physiological activity in response to a variety of external stimuli 2. Secondary metabolites include polysaccharides, lectins, terpenoids, alkaloids, and metal chelators 3. These compounds exhibit anticancer properties, immunomodulatory, antibacterial, antidiabetic and anti-HIV properties 3. Among the characteristics, much attention is focused on the anticancer effect of secondary metabolites, and many clinical trials are underway to evaluate its efficacy and safety 4. In a phase II clinical trial, *Ganoderma lingzhi*, a reishi mushroom, proved successful in preserving quality of life in cancer patients following chemotherapy 5. Reishi mushroom also acted as an immunomodulatory agent to increase the expression of IL-2, IL-6 and IFN-γ, and was effective in orally administered colorectal cancer patients 6. Furthermore, in phase 1 clinical trial, *Agaricus bisporus*, the white button mushroom, demonstrated promising outcomes in the treatment of patients with recurrent prostate cancer by suppressing the prostate-specific antigen 7. Based on these findings, it will be worthwhile to discover more new mushrooms with anticancer activity and apply their efficacy to the treatment of cancer patients.

Mushrooms have a unique life cycle consisting mainly of short-lived fruiting bodies and long-lived mycelium 8. The mycelium acts as the root of the plant and has a network-like structure with roots in the ground and extending branches. This cobweb-like system becomes the basic structure for fungi to produce mushrooms. Recently, a liquid culture method of mycelium has been developed for the mass production 9. The advantage of mycelium liquid culture is that a large amount of mycelium can be produced at a low production cost due to the rapid colonization rate 10. Mycelium liquid culture is considered a promising system for the efficient production of mycelium-derived secondary metabolites because it can meet current good manufacturing practice (cGMP) requirements for the production of bioactive reagents.

One of the crucial problems in cancer treatment is the development of chemo-resistant cells 11. Although conventional chemotherapy improved 5-year survival rates, the overall mortality rate for cancer patients did not change significantly because most patients eventually relapse and eventually die from cancer 12. Numerous studies are being conducted to elucidate novel mechanisms to treat anticancer resistance. Recently, side population (SP) cells have been shown to promote cancer recurrence while exhibiting anticancer resistance during chemotherapy 13, 14. SP cells are a subpopulation of cells that derive from differentiated cells in the early stages of tumors and have a special ability to induce tumorigenesis and carcinogenesis 13, 14. Eventually, SP cells retain all types of malignancies, leading to tumor recurrence.

*Polyporus* is a wood-rotting mushroom and is a genus of *Poroid* in the family *Polyporaceae* 15. Recently, *Polyporus umbellatus* was found to inhibit tumor cell proliferation and promote apoptosis 16. However, no anticancer effects were reported in other species belonging to *Polyporus*. The aim of this study was to investigate the anticancer effect of mycelium culture extracts of various *Polyporus* species. Here, we identified the mycelium culture extract showing anticancer property and the active ingredient of the extract.

## Materials and Methods

### Mycelium liquid culture

*Polyporus arcularius* (Accession number: NIBRFGC000143418), *Polyporus brumalis* (Accession number: NIBRFGC000500533) and *Polyporus parvovarius* (Accession number: NIBRFGC000500557) were obtained from National institute of biological resources (NIBR, Incheon, Korea). Four different media were used as follows: DY (dextrose (215530; BD Difco, Franklin, NJ, USA) 20 g/l and yeast extract (212750; BD Difco) 2g/l), MEB (maltose (216830; BD Difco) 1.8 g/l, dextrose (215530; BD Difco) 6 g/l, malt extract (218630; BD Difco) 6 g/l, and yeast extract (212750; BD Difco) 2g/l), MY (malt extract (218630; BD Difco) 20 g/l and yeast extract (212750; BD Difco) 2g/l), and PDB (dextrose (215530; BD Difco) 20 g/l and Potato starch (S2004; Sigma, Saint Louis, MO, USA) 4 g/l). The procedure for preparing the extract from the mycelium culture was as previously described 17.

### Cell Culture

HeLa cells (A1100001; Thermo Fisher Scientific, Waltham, MA, USA), BEAS-2B (CRL-9609™; American Type Culture Collection, Manassas, VA, USA), NCI-H1299 (CRL-5803™; American Type Culture Collection), HCC15 (ACC 496; Deutsche Sammlung von Mikroorganismen und Zellkulturen, Braunschweig, German), HCC95 (70095; Korean Cell Line Bank, Seoul, Korea), A549 (CRL-185™; American Type Culture Collection), PC-9 (90071810; European Collection of Authenticated Cell Cultures, Salisbury, UK), and NCI-H2009 (CRL-5911™; American Type Culture Collection) were used in this study. Cells culture was performed as described previously 18.

### Cell proliferation assay

96-well plates were used for cell growth at a density of 2,000 cells per well. Each extract from mycelium liquid culture was diluted at concentrations of 0.1 mg/ml. Paclitaxel (T7191-1MG; Sigma) was diluted to a final concentration of 40 nM. A cell proliferation assay based on a DNA content-based technique was utilized to precisely count the number of cells 19. Cell proliferation was measured on the fourth day after drug treatment.

### Determination of cytotoxicity of pp-DY and PCA

96-well plates were used for cell growth at a density of 2,000 cells per well. pp-DY was diluted at concentrations of 0.1 to 0.5 mg/ml. 3,4-dihydroxybenzaldehyde (Protocatechualdehyde, PCA, D108405-5G; Sigma) was diluted at concentrations of 50 to 200 μg/ml. Cell proliferation was measured on the fourth day after drug treatment. Cell viability was determined by measuring cell proliferation at each concentration and normalizing the proliferation values to those in DMSO.

### Determination of the optimal concentration of pp-DY

96-well plates were used for cell growth at a density of 2,000 cells per well. Cell proliferation was assessed at 24, 48, 72, and 96 h at doses of 0.1 to 0.5 mg/ml in order to determine the optimal concentration of pp-DY that might inhibit cell proliferation.

### Apoptosis assay

Apoptosis assay using the FITC Annexin V Apoptosis Detection Kit (556547; BD Biosciences, Franklin Lakes, NJ, USA) was performed as previously described 20.

### Cell cycle assay

Cell cycle assay was performed as previously described 21. In brief, 1 × 10^6^ cells were collected by centrifugation at 400 × *g* for 2 min. Then, cells were fixed with 70% EtOH and stained with 50 μg/ml propidium iodide (PI, P4170-10MG; Sigma).

### Western blot analysis

Gel electrophoresis and transfer was performed as described previously 18. Using a Chemidoc XRS+ system (1708265; BIO-RAD; Hercules, CA, USA), proteins were detected with SuperSignal™ West Femto chemiluminescent solution (34095; Thermo Fisher Scientific, Waltham, MA, USA). Primary antibodies used in this study included the cleaved caspase 9 antibody (52873; 1:500 dilution; Cell signaling technology, Danvers, MA, USA), the cleaved caspase 3 antibody (9664; 1:500 dilution; Cell signaling technology), p16INK4A antibody (sc-1661; 1:500 dilution; Santa Cruz Biotechnology, Dallas, TX, USA), p21Cip1 antibody (sc-6246; 1:500 dilution; Santa Cruz), phospo-ERK antibody (9101s; 1:500 dilution; Cell signaling technology, Danvers, MA, USA) and HRP-conjugated β-actin (sc47778; 1:1000 dilution; Santa Cruz). Secondary antibodies included HRP-conjugated anti-mouse antibody (sc-516102; 1:1000 dilution; Santa Cruz) and HRP-conjugated anti-rabbit antibody (sc-2357; 1:1000dilution; Santa Cruz).

### Fractionation and structural analysis of the extract from *Polyporus parvovarius* mycelium cultured in DY medium (pp-DY)

Fractionation and structural analysis of pp-DY were performed in our previous study 17.

### Side population (SP) assay

The proportion of SP cells was evaluated using media containing 100 μM PCA or 20 μM cisplatin (PHR1624; Sigma). Briefly, 1 × 10^6^ cells were treated with 5 μg/ml Hoechst 33342 (H3570; Thermo Scientific, Waltham, MA, USA) or 5 μM verapamil (V4629; Sigma) at 37°C for 90 min. After washing with PBS, cells were stained with 1 μg/ml PI on ice for 5 min. Flow cytometry analysis was performed by using LSR II (BD Biosciences).

### Colony formation assay

Soft agar assays were performed according to the manufacturer-supplied protocol (ECM570; Millipore, Burlington, MA, USA).

### Statistical analyses

A common statistical software application was used to conduct the statistical analysis (SigmaPlot 12.5; Systat Software, San Jose, CA, USA). To ascertain whether differences were significant, a student's t-test or two-way ANOVA followed by Bonferroni post hoc-test was used.

## Results

### Chemical screening of *Polyporous* mycelium culture extracts with anticancer properties

**Figure 1:**
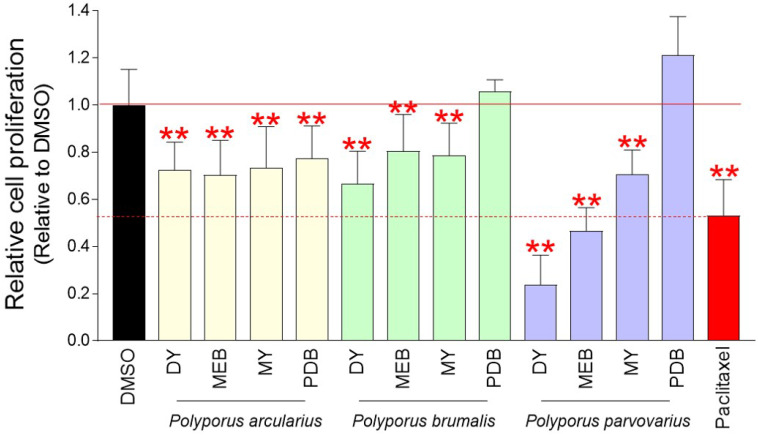
** Chemical screening of *Polyporous* mycelium culture extracts with anti-cancer properties.** Twelve extracts were obtained from three different *Polyporus* species (*Polyporus arcularius*, *Polyporus brumalis*, *Polyporus parvovarius*) that were cultured in four different culture media (DY, MEB, MY, and PDB). Each extract diluted to a concentration of 0.1 mg/ml in the medium was added to Hela cells, and the cell proliferation inhibitory effect was measured on the fourth day. Paclitaxel was used as a positive control group. **P < 0.01, student t-test. Means ± S.D., *n* = 10.

A screening strategy was used to find *Polyporous* mycelium culture extracts that significantly inhibit the growth of Hela cells derived from cervical cancer. The cell number was determined using a DNA content-based approach 19. Twelve extracts were obtained from three different *Polyporus* species that were cultured in four different culture media (DY, MEB, MY, and PDB). Each extract diluted to a concentration of 0.1 mg/ml was added to Hela cells, and the cell proliferation inhibitory effect was measured on the fourth day. As a positive control group, paclitaxel, which is frequently used to treat a number of cancers including ovarian cancer, breast cancer, and pancreatic cancer, was utilized 22. Mycelium culture extracts of *Polyoorus arcularius* in DY, MEB, MY and PDB media significantly inhibited cancer cell proliferation compared to DMSO control (Fig. 1). Moreover, mycelium culture extracts of *Polyoorus brumalis* cultured in DY, MEB, MY and PDB media significantly inhibited cancer cell proliferation compared to DMSO control (Fig. 1). Finally, mycelial culture extracts of *Polyporus parvovarius* cultured in DY, MEB and MY media showed significant cancer cell suppression ability compared to DMSO control (Fig. 1). The more antiproliferative effect than paclitaxel was *Polyporus parvovarius* mycelium culture extract cultured in DY or MEB medium (Fig. 1). Since the mycelium culture extract of *Polyporus parvovarius* in DY medium (a.k.a., pp-DY) showed the most antiproliferative effect, pp-DY was chosen for further analysis.

**Figure 2:**
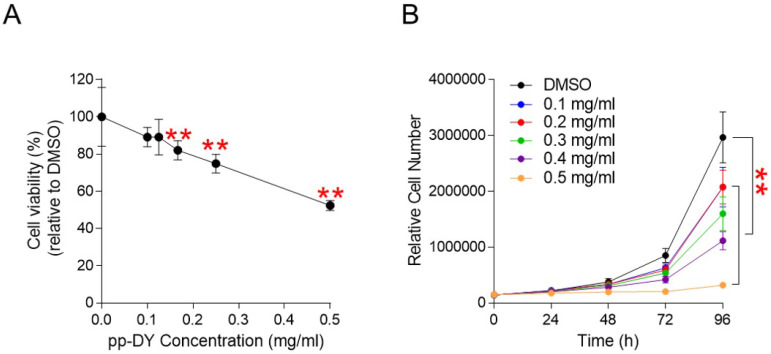
** Determination of cytotoxicity and optimal concentration of pp-DY. (A)** Cell viability was determined by measuring cell proliferation at pp-DY concentrations of 0.1-0.5 mg/ml and normalizing the proliferation values to those in DMSO. **P < 0.01, student t-test. Means ± S.D., *n* = 10. **(B)** Cell proliferation was assessed at 24, 48, 72 and 96 h after treatment at concentrations of 0.1 to 0.5 mg/ml. **P < 0.01, two-way ANOVA followed by Bonferroni post hoc-test. Mean ± S.D., *n* = 10.

Although pp-DY exhibited a cancer cell proliferation inhibitory effect, the possibility that this effect was due to the cytotoxicity rather than the anticancer effect could not be excluded. To exclude the possibility, cell viability was measured at a pp-DY concentration of 0.1-0.5 mg/ml. The reduction in cell viability was concentration-dependent, indicating that the cell proliferation inhibitory effect of pp-DY was not due to the cytotoxicity of pp-DY (Fig. 2A).

We then investigated the optimal pp-DY concentration that could effectively reduce cell proliferation. Cell proliferation was assessed at 24, 48, 72 and 96 h after treatment at concentrations of 0.1 to 0.5 mg/ml. Compared to the DMSO control, each dose demonstrated a significant reduction in cell proliferation (Fig. 2B). However, the concentration of pp-DY at 0.5 mg/ml showed the greatest decrease compared to other concentrations (Fig. 2B). Therefore, the concentration of pp-DY diluted to 0.5 mg/ml was selected for further analysis and applied to all subsequent experiments.

### pp-DY inhibits cancer cell proliferation by inducing apoptosis and S-phase arrest

**Figure 3:**
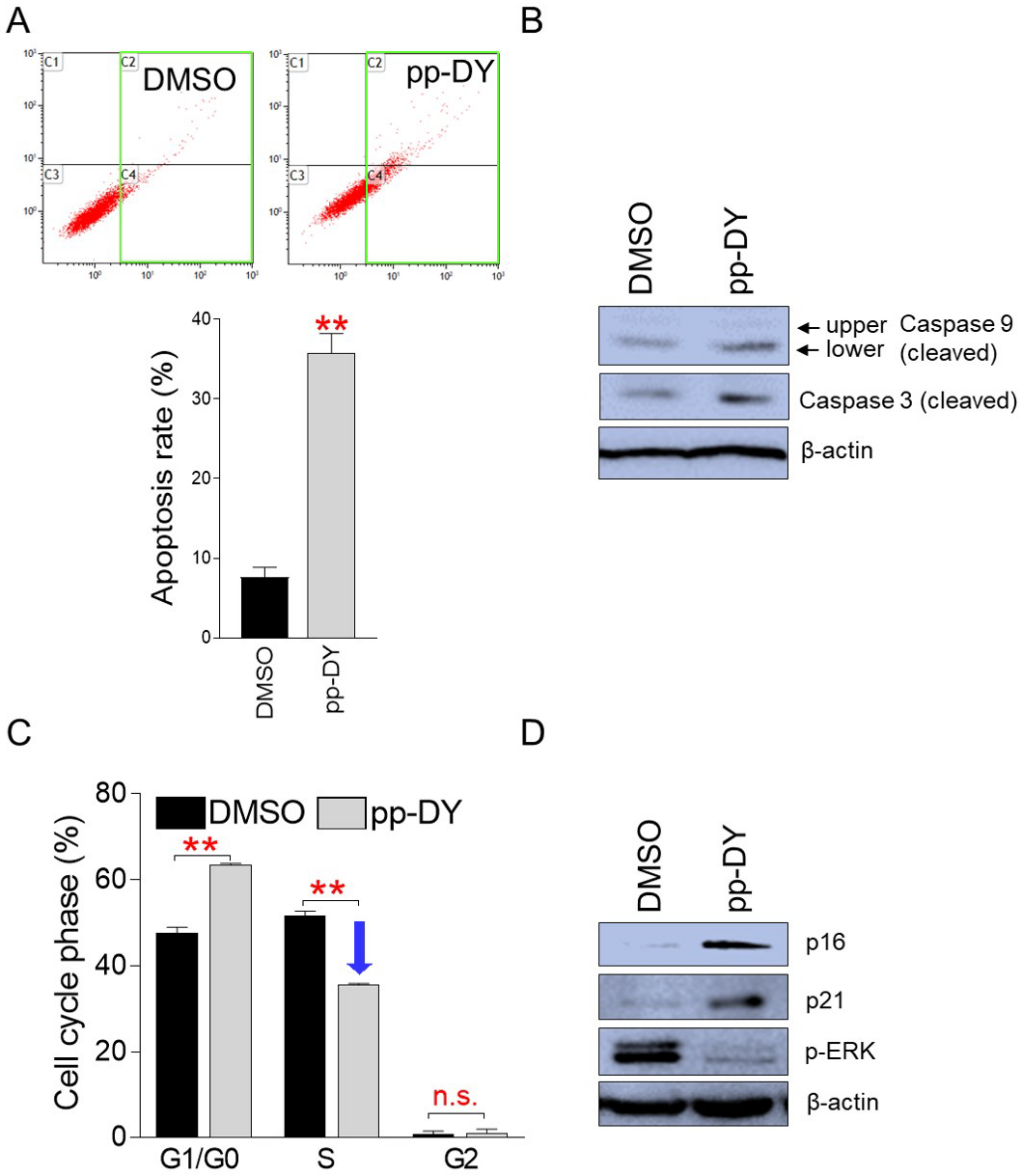
** pp-DY inhibits cancer cell proliferation by inducing apoptosis and S-phase arrest. (A)** Flow cytometric analysis of apoptotic cells was performed by using annexin V staining. Green square indicates populations of apoptotic cells. **P < 0.01, student's t-test. Means ± S.D., *n* = 3. **(B)** Effect of PCA on the expression level of proteins involved in apoptosis pathway. Cleaved form of caspase 9 and caspase 3. Full gels are shown in Supplementary Fig. 2. **(C)** Flow cytometric analysis of cell cycle was performed by using PI staining. **P < 0.01, student's t-test. Means ± S.D., *n* = 3. **(D)** Effect of PCA on the expression level of proteins involved in cell cycle pathway. p16INK4A (p16), p21Cip1 (p21) and phopho-ERK (p-ERK). Full gels are shown in Supplementary Fig. 2.

Controlling the uncontrolled growth of cancer cells using apoptosis is known as an effective way to treat cancer 23. Therefore, we investigated whether inhibition of cancer cell growth by pp-DY is facilitated by apoptosis. Indeed, the proportion of apoptotic cells by pp-DY treatment was significantly increased compared to DMSO control (Fig. 3A). These results indicate that pp-DY inhibits the growth of cancer cells by inducing apoptosis.

To further support the induction of apoptosis by pp-DY, we examined the cleavage of caspase 9, which functions as an initiator apoptosis, and caspase 3, which functions as an effector apoptosis 24. The lower cleaved form of caspase 9 was markedly increased in pp-DY-treated group compared to that in DMSO control, indicating increased caspase cascade initiation in the pp-DY group (Fig. 3B). Furthermore, the cleavage of caspase 3 was markedly increase in pp-DY group compared to that in DMSO control, indicating increased execution of apoptosis in the pp-DY group (Fig. 3B).

Cancer is also characterized by uncontrolled proliferation due to the abnormal activity of various cell cycle proteins 25. Cell cycle arrest, especially S-phase arrest, functions as one of important indicators to confirm anticancer activity 26, 27. Therefore, we investigated whether the inhibition of cancer cell growth by pp-DY was due to S-phase arrest. Cells treated with pp-DY significantly increased the proportion of G1/G0 phase from 47.6% to 63.4% compared to DMSO control (Fig. 3C). Furthermore, cells treated with pp-DY significantly reduced the proportion of S phase from 51.3% to 35.4% compared to DMSO control, suggesting that pp-DY inhibits the growth of cancer cells by inducing S-phase arrest (Fig. 3C).

Cell cycle pathways are regulated by several proteins, including p16INK4A (p16) and p21Cip1 (p21). Therefore, we investigated how the expression levels of these proteins were affected by pp-DY treatment. Expression of p16 and p21 was significantly increased by pp-DY treatment, confirming pp-DY mediated S-phase arrest at the protein level (Fig. 3D). In addition, since the extracellular signal-regulated kinase (ERK) of MAP kinases is an important regulator of S-phase entry, the expression level of an activated form of ERK, phospho-ERK, was investigated. Expression of phospho-ERK decreased upon pp-DY treatment, indicating a pp-DY-mediated decrease in S-phase entry (Fig. 3D). These results confirmed that pp-DY suppressed the proliferation of cancer cells by inducing S-phase arrest.

### Anticancer effect of pp-DY on various lung cancer-derived cells

**Figure 4:**
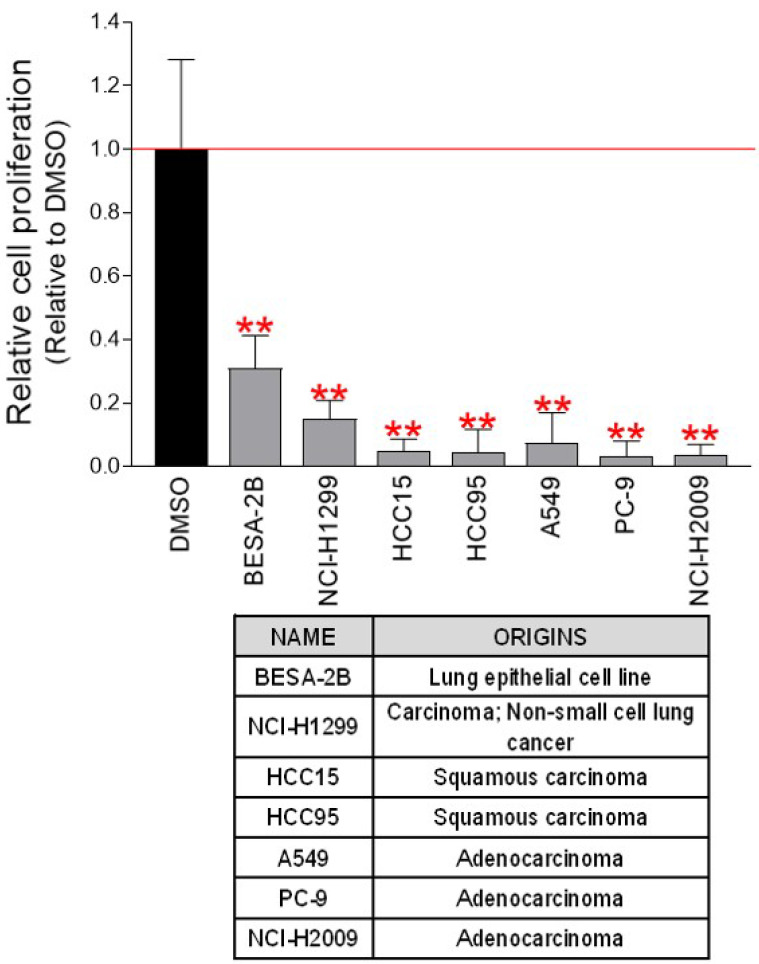
** Anticancer effect of pp-DY on various lung cancer-derived cells.** pp-DY diluted to a concentration of 0.5 mg/ml in the medium was added to various lung cancer-derived cells, and the cell proliferation inhibitory effect was measured on the fourth day. **P < 0.01, student t-test. Means ± S.D., *n* = 10.

To investigate the potential anticancer efficacy of pp-DY, Hela cell was used. We then investigated whether the anticancer efficacy of pp-DY was effective not only in Hela cells but also in cell lines derived from other cancer types. The chemotherapeutic impact of pp-DY was examined using seven cancer cell lines derived from lung cancer. As seen in Hela cells, pp-DY significantly reduced cancer cell proliferation in all cell lines tested (Fig. 4). These results show that the anticancer properties of pp-DY can be applied to numerous types of cancer cell lines, and open the possibility of using pp-DY for the treatment of various cancer cells.

### Identification of active ingredients of pp-DY through fractionation and structural analysis

**Figure 5:**
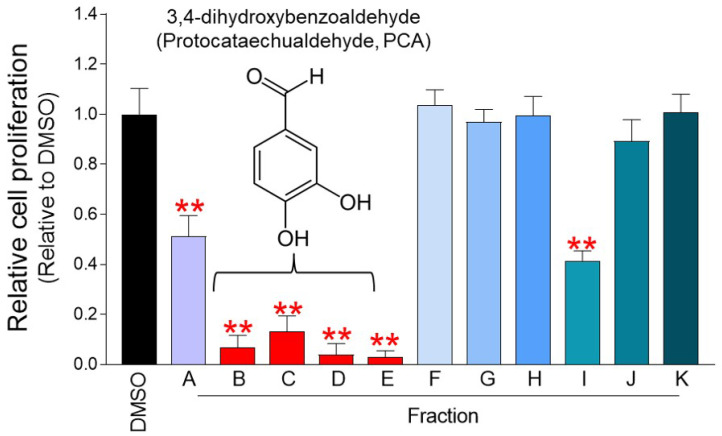
** Identification of active ingredients of pp-DY through fractionation and structural analysis.** Eleven fraction samples from A to K were obtained and their effect on cell proliferation in Hela cells was investigated. The cell proliferation inhibitory effect was measured on the fourth day after treatment. **P < 0.01, student t-test. Means ± S.D., *n* = 10. Fractions from B to E were analyzed using ^1^H NMR, ^13^C NMR, ^1^H-^1^H COSY spectrum, and HSQC/HMBC spectrum structural analysis 17. Structural analysis revealed that fractions from B to E were 3,4-dihydroxybenzaldehyde (Protocatechualdehyde, PCA) 17.

pp-DY is a mycelium culture extract containing various active ingredients, but it is not known which of them exhibits an anticancer effect. Therefore, in order to find the active ingredient of the extract, the fractionation process was performed using Diaion HP-20 column chromatography, silica gel column chromatography, and Sephadex LH-20 column chromatography in order 17. Eleven fraction samples from A to K were obtained and their effect on cell proliferation was investigated. Among the 11 fractions, the fractions that inhibited the proliferation the most were fractions B, C, D, and E (Fig. 5). In our previous study, fractions from B to E were analyzed using ^1^H NMR, ^13^C NMR, ^1^H-^1^H COSY spectrum, and HSQC/HMBC spectrum structural analysis 17. Structural analysis revealed that fractions from B to E were 3,4-dihydroxybenzaldehyde (Protocatechualdehyde, PCA) (Fig. 5) 17.

### PCA is an active ingredient of pp-DY showing anticancer properties

**Figure 6:**
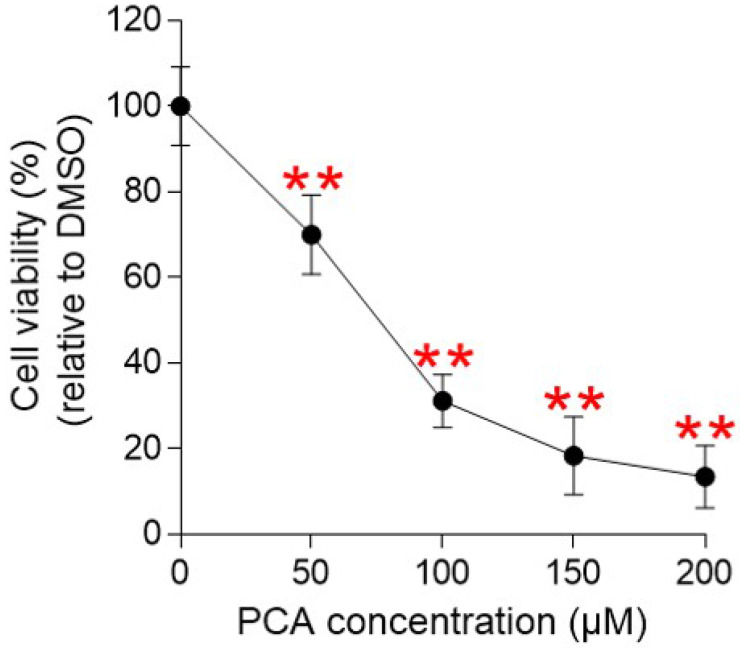
** Determination of cytotoxicity of PCA.** Cell viability was determined by measuring cell proliferation at PCA concentrations of 50-200 μM and normalizing the proliferation values to those in DMSO. **P < 0.01, student t-test. Means ± S.D., *n* = 10.

The identification of PCA as an active ingredient exhibiting anticancer efficacy led to the investigation of whether PCA had anticancer activity similar to that observed in pp-DY. Before confirming the anticancer activity of PCA, we investigated whether PCA was cytotoxic. Cell viability was measured after Hela cells were treated with PCA at a concentration of 50-200 µM (Fig. 6). The cell viability decreased in a PCA concentration-dependent manner, suggesting that the cell proliferation inhibitory effect of PCA was not due to the cytotoxicity of PCA (Fig. 6). Therefore, the concentration of PCA diluted to 100 µM was selected for further analysis and applied to all subsequent experiments.

**Figure 7:**
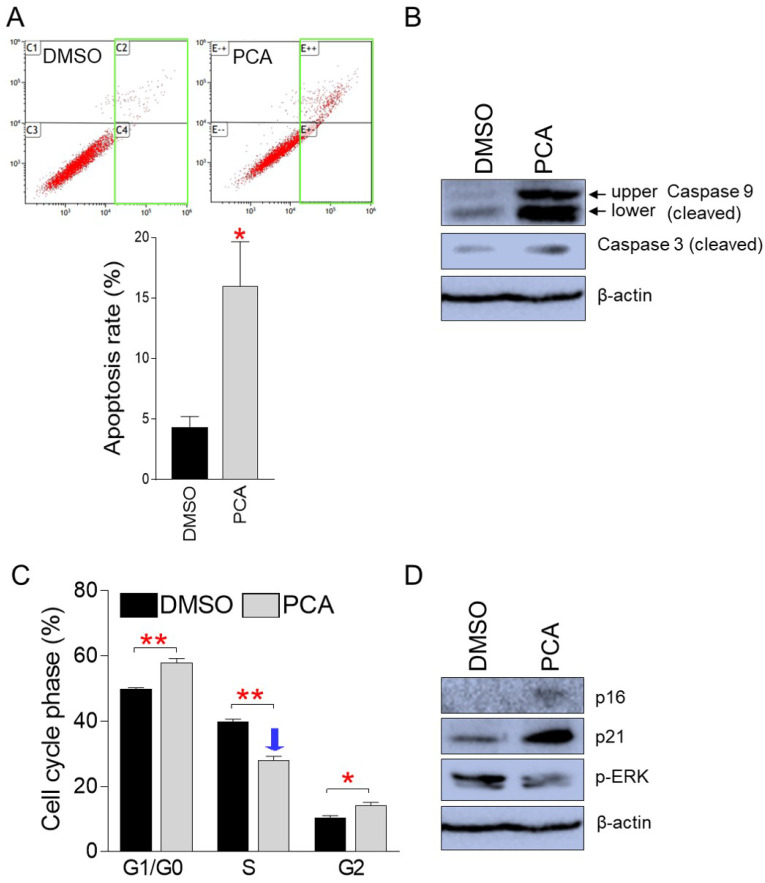
** PCA inhibits cancer cell proliferation by inducing apoptosis and S-phase arrest. (A)** Flow cytometric analysis of apoptotic cells was performed by using annexin V staining. Green square indicates populations of apoptotic cells. **P < 0.01, student's t-test. Means ± S.D., *n* = 3. **(B)** Effect of PCA on the expression level of proteins involved in apoptosis pathway. Cleaved form of caspase 9 and caspase 3. Full gels are shown in Supplementary Fig. 2. **(C)** Flow cytometric analysis of cell cycle was performed by using PI staining. **P < 0.01, student's t-test. Means ± S.D., *n* = 3. **(D)** Effect of PCA on the expression level of proteins involved in cell cycle pathway. p16INK4A (p16), p21Cip1 (p21) and phopho-ERK (p-ERK). Full gels are shown in Supplementary Fig. 2.

We then investigated whether the PCA-mediated inhibition of cancer cell proliferation is due to apoptosis. As demonstrated in pp-DY, PCA treatment significantly increased the proportion of apoptotic cells compared to DMSO control, indicating that PCA also suppresses the proliferation of cancer cells by inducing apoptosis (Fig. 7A).

To further support the induction of apoptosis by PCA, we examined the cleavage of caspase 9 and caspase 3. The upper and lower cleaved form of caspase 9 was markedly increased in PCA-treated group compared to that in DMSO control, indicating increased caspase cascade initiation in the PCA group (Fig. 7B). Furthermore, the cleavage of caspase 3 was markedly increase in PCA-treated group compared to that in DMSO control, indicating increased execution of apoptosis in the PCA group (Fig. 7B).

Cell cycle analysis was also performed for the same purpose. As demonstrated in pp-DY, PCA treatment significantly increased the proportion of G1/G0 phase from 49.8% to 57.9% compared to the DMSO control (Fig. 7C). In addition, PCA treatment significantly reduced the proportion of S-phase from 39.9% to 28.0% compared to the DMSO control, suggesting that PCA also inhibits the growth of cancer cells by causing S-phase arrest (Fig. 7C).

We then investigated how the expression levels of cell cycle regulator were affected by PCA treatment. Expression of p16 and p21 was significantly increased by PCA treatment, confirming PCA-mediated S-phase arrest at the protein level (Fig. 7D). Furthermore, expression of phospho-ERK decreased upon PCA treatment, indicating a PCA-mediated decrease in S-phase entry (Fig. 7D). These results confirmed that PCA suppressed the proliferation of cancer cells by causing S-phase arrest.

**Figure 8:**
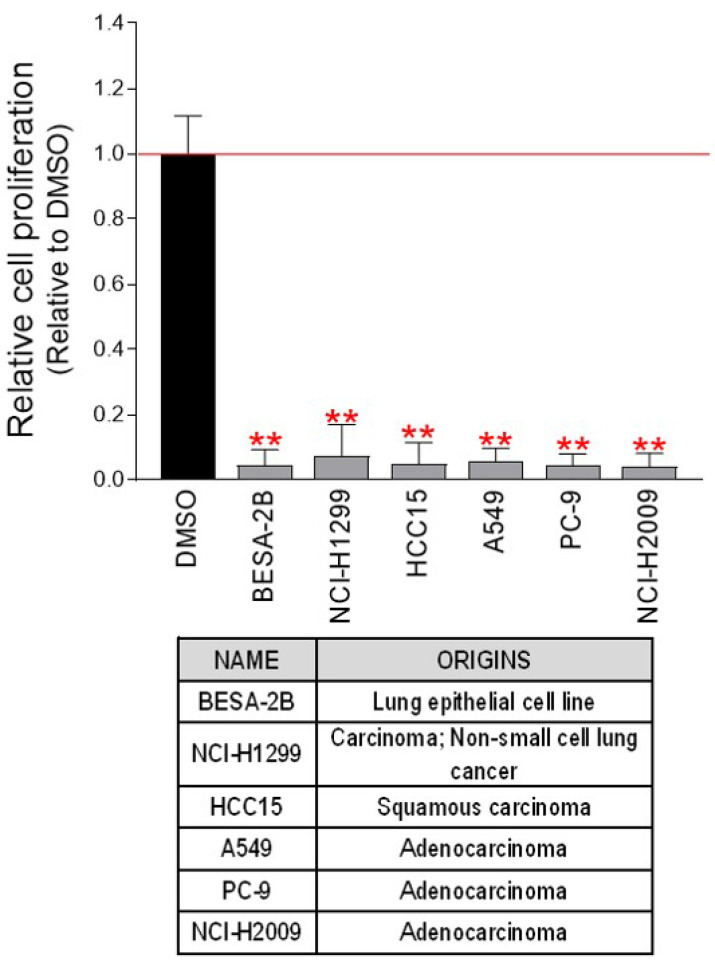
** Anticancer effect of PCA on various lung cancer-derived cells.** PCA diluted to a concentration of 100 μM in the medium was added to various lung cancer-derived cells, and the cell proliferation inhibitory effect was measured on the fourth day. **P < 0.01, student t-test. Means ± S.D., *n* = 10.

We then investigated whether the anticancer efficacy of PCA was effective not only in Hela cells but also in cancer cell lines derived from other cancers. The chemotherapeutic impact of PCA was examined using six cancer cell lines derived from lung cancer. As seen in Hela cells, PCA significantly reduced cell proliferation in all cell lines tested (Fig. 8). These results show that the anticancer properties of PCA are not limited to specific cancer cell lines, and open the possibility of using PCA for the treatment of various cancer cells.

Taken together, PCA exhibits anticancer effects similar to those of pp-DY, suggesting that PCA is an active component of pp-DY exhibiting anticancer effects.

### PCA does not have side effects such as increased proportion of side population (SP) cells

**Figure 9:**
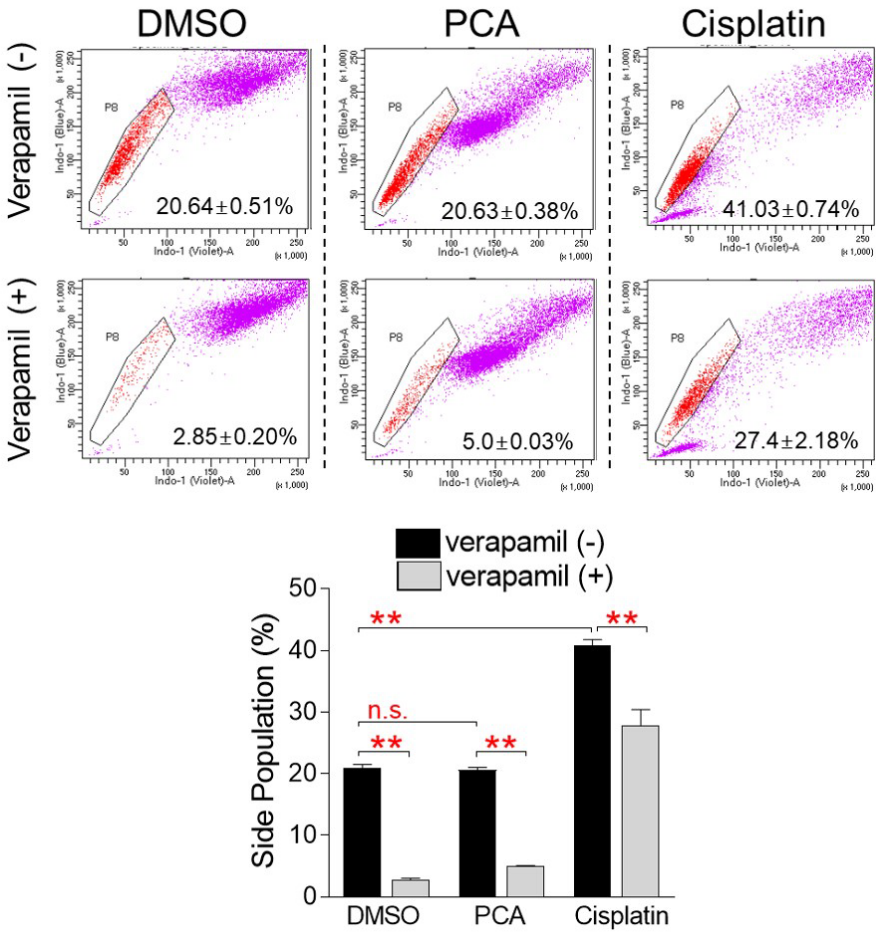
** PCA exhibits no side effects such as increased SP cell proportion.** Cisplatin was used as a positive control to evaluate the effect of PCA on the chemoresistance of cancer cells. Cells were stained with Hoechst 33342. Verapamil was used to inhibit the efflux of Hoechst 33342. Analysis of side population was performed by using flow cytometry analysis. The pentagon gate was used to identify the side population in flow chart. **P < 0.01, n.s. (not significant), student t-test. Means ± S.D., *n* = 3.

The emergence of chemo-resistant caner is one of the key problems with cancer treatment 11. The properties of cells that are particularly resistant to chemotherapeutic treatments are shared by side population (SP) cells 28. Therefore, when SP cells are stained with Hoechst 33342 dye, they rapidly release the dye through the ATP binding cassette (ABC) transporter and are characterized as unstained compared to other cells 29. However, when SP cells are treated with inhibitors of the ABC transporter such as verapamil, they are unable to efflux the dye through the ABC transporter, resulting in loss of their properties 29. Cisplatin is the most widely used anticancer drug and is very effective in the treatment of many types of cancer, but like other chemotherapeutic agents, it has fatal problems such as drug resistance through increasing the proportion of SP cells 30. We then investigated whether PCA, like other chemotherapeutic drugs, caused problems such as increased SP cell generation. In the DMSO control group, the proportion of SP cells was chosen to be 20.64% of the total cells (Fig. 9). When the DMSO control group was treated with verapamil, the proportion of SP cells significantly decreased to 2.9%, indicating that the selected cells had the characteristics of SP cells (Fig. 9). The proportion of SP cells in the PCA-treated group was selected to be 20.63% of the total cells, which was similar to the DMSO-treated group, indicating that PCA does not exhibit side effects such as increased SP cell proportion seen in other anticancer drugs (Fig. 9). When the PCA-treated group was treated with verapamil, the proportion of SP cells significantly decreased to 5.0%, indicating that the selected cells had the characteristics of SP cells (Fig. 9). However, the proportion of SP cells in the cisplatin-treated group was selected to be 41.03% of the total cells, which was significantly higher than that in the DMSO and PCA groups (20.64% and 20.63%, respectively), indicating that cisplatin increased the proportion of SP cells seen in other anticancer drugs (Fig. 9). When verapamil was applied in the cisplatin-treated group, the proportion of SP cells was significantly reduced to 27.4%, indicating that the selected cells had the characteristics of SP cells (Fig. 9). Taken together, these results indicate that cisplatin, one of the widely used anticancer drugs, affected the chemoresistance of cancer cells by increasing the proportion of SP cells, but PCA, the active ingredient of *Polyporus parvovarius* mycelium extract, did not.

Stemness features such as tumor sphere formation capacity is the main characteristics of SP cells 31. Thus, we investigated the effect of PCA and pp-DY on tumor sphere formation. The capacity of tumor sphere formation was assessed by using soft agar assay. While DMSO control group formed colonies as an indicator of tumor sphere formation, PCA and pp-DY-treated groups failed to form colonies (Supplementary Fig. 1). These results indicate that PCA and pp-DY did not affect stemness features such as tumor sphere formation.

## Discussion

Mushrooms synthesize secondary metabolites, bioactive compounds, including polysaccharides, steroids, terpenes and peptides 32. These metabolites have many medicinal properties, such as anti-tumor and antioxidant 32. Because secondary metabolites of mushrooms are non-toxic and have few or no side effects, their medicinal potential takes precedence over other natural compounds 33. In this study, the anticancer effect of extracts from three *Polyporus* mycelium cultured in four different media (DY, MEB, MY, PDB) was investigated. The extract of *Polyporus parvovarius* mycelium cultured in the DY medium exhibited anticancer activity, indicating that the mycelium produced effective secondary metabolites using the DY medium. However, the extract of *Polyporus parvovarius* mycelium cultured in PDB medium did not show anticancer activity, suggesting that PDB medium is not optimal for synthesizing secondary metabolites that exhibit anticancer properties. This phenomenon is explained by the observation that the mycelium of each mushroom requires an optimal source of carbon and nutrients to produce secondary metabolites 34. DY and PDB medium contain equal amounts of glucose, whereas DY medium contains only yeast extract and PDB medium contains potato starch instead of yeast extract. Considering the difference in medium composition, the medium containing yeast extract plays the most important role in the production of secondary metabolites exhibiting anticancer effects in *Polyporus parvovarius*. To the best of our knowledge, this is the first report showing optimal media conditions for *Polyporus parvovarius* mycelium for synthesizing secondary metabolites with anticancer activity. If the composition of DY medium is further optimized through the procedure such as spent medium analysis, it will be possible to optimize a medium composition in which *Polyporus parvovarius* mycelium can more efficiently produce secondary metabolites with anticancer properties.

Chemotherapy is one of the main anticancer therapies and is very effective in killing rapidly dividing cancer cells 35. However, although chemotherapy is effective in cancer treatment in the early stages, it becomes ineffective as cancer cells develop chemoresistance 36. For example, paclitaxel, used as a form of chemotherapy, kills cancer cells in the early stages of chemotherapy, but not eventually because of drug resistance 37. Therefore, the identification of secondary metabolites that may not induce drug resistance in mushroom will open new paradigm in cancer therapeutics. Recent studies have shown that the cause of drug resistance in cancer is the persistence of SP cells 38, 39. Drug efflux pumps, such as ABC transporters, ABCG2 or ABCB1, are activated in SP cells and help to efflux various cancer drugs, thereby generating a multidrug resistance phenotype. Therefore, SP cells have received the most clinical attention as a major cause of drug resistance during chemotherapy and tumor recurrence 40, 41. In the present study, we first identified PCA as a secondary metabolite with anticancer activity in *Polyporus parvovarius* mycelium extract. This novel finding was evidenced by the results that PCA exhibited the same anticancer properties as *Polyporus parvovarius* mycelium extract. Furthermore, the novelty of our study is supported by the finding that PCA did not increase the proportion of SP cells, unlike conventional anticancer drugs. Although further studies are needed to elucidate the underlying mechanisms by which PCA did not increase the proportion of SP cells, we propose that PCA alone or in combination with other cancer drugs will be effective in patients developing drug resistance.

Apoptosis is a mechanism by which eukaryotic cells commit suicide without causing an unintended inflammatory response 42. Since apoptosis evasion is a hallmark of cancer, modulation of apoptosis has been used as an effective method to prevent the uncontrolled growth of cancer cells 23. Indefinite cell cycle progression is a phenomenon observed in cancer cells due to abnormalities in regulatory proteins involved in the cell cycle 25. Therefore, as with normal cells that can only pass through a limited cell cycle, cell cycle control has been considered a major therapeutic goal for cancer. Here, pp-DY and its active ingredient, PCA, effectively killed cancer cells by inducing apoptosis and S-phase arrest. These beneficial effects were not limited to specific cancer cells, but effectively killed various types of cancer cells. Since the induction of apoptosis and S-phase arrest by these agents acts like a double-edged sword in killing cancer cells, we suggest that pp-DY and PCA should be prioritized as effective therapeutic options for the treatment of various types of cancer.

Mycelium liquid culture is a novel method for growing mycelium in liquid without the use of traditional agar plates 43. Compared to the solid medium-based culture that has been widely used so far, the mycelium liquid culture has the advantage of requiring less production time and culture space 44. Furthermore, it enables rapid colonization of the mycelium, allowing large-scale expansion of culture and mass production of secondary metabolites at low cost 9. Therefore, the mycelium liquid culture has been used for the production of carbohydrates and polysaccharides, which are considered prebiotics as functional foods, and is recently used for the production of high value-added biologics such as enzymes and pharmaceuticals 44. Here, we cultured *Polyporus parvovarius* mycelium in DY medium to produce secondary metabolites with anticancer effects. However, the amount of secondary metabolites obtained in a single culture was limited because the culture was performed within an acceptable volume on a laboratory scale. If we optimize the mass culture production process by adjusting process variables such as pH, temperature, dissolved oxygen, and agitation speed, we can maximize the amount of secondary metabolites generated during *Polyporus parvovarius* mycelium culture.

In summary, secondary metabolites exhibiting anticancer activity were found in the extract of *Polyporus parvovarius* mycelium, and PCA was confirmed as an active ingredient of anticancer activity. Both pp-DY and PCA inhibited cancer cell proliferation by inducing apoptosis and S-phase arrest. Furthermore, unlike conventional anticancer drugs, PCA did not increase the proportion of SP cells, suggesting that PCA can be used to treat drug-resistant cancer. Taken together, our research revealed the mycelium culture extract exhibiting anticancer properties and its active ingredient. These novel anticancer substances can be produced at low cost through mass culture of mycelium, and have a clinical use in the treatment of cancer and recurrent cancer.

## Supplementary Material

Supplementary figures.
