# Supplementary material for: Chemical screening identifies the anticancer properties of Polyporous parvovarius
Source: J Cancer. 2023 Jan 1;14(1):50–60. doi: 10.7150/jca.78302 (PMC9809329; doi:10.7150/jca.78302)

Supplementary Fig. 1. The effect of PCA and pp-DY on stemness features such as tumor sphere formation. Scale bar 20 μm.

Supplementary Fig. 2. Full-size image of Western blot in Fig. 3B, 3D, 7B and 7D.

Supplementary Fig. 1

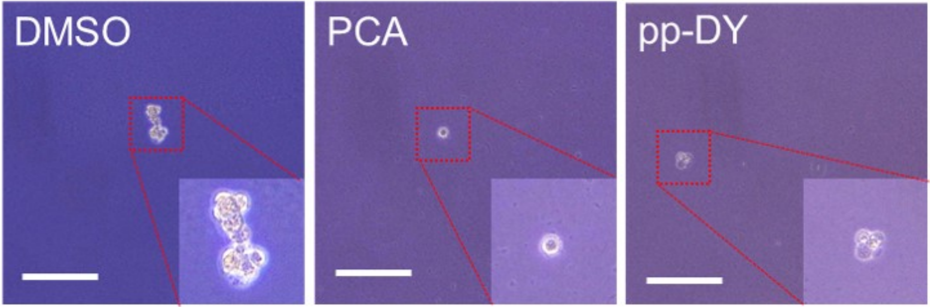

Supplementary Fig. 2

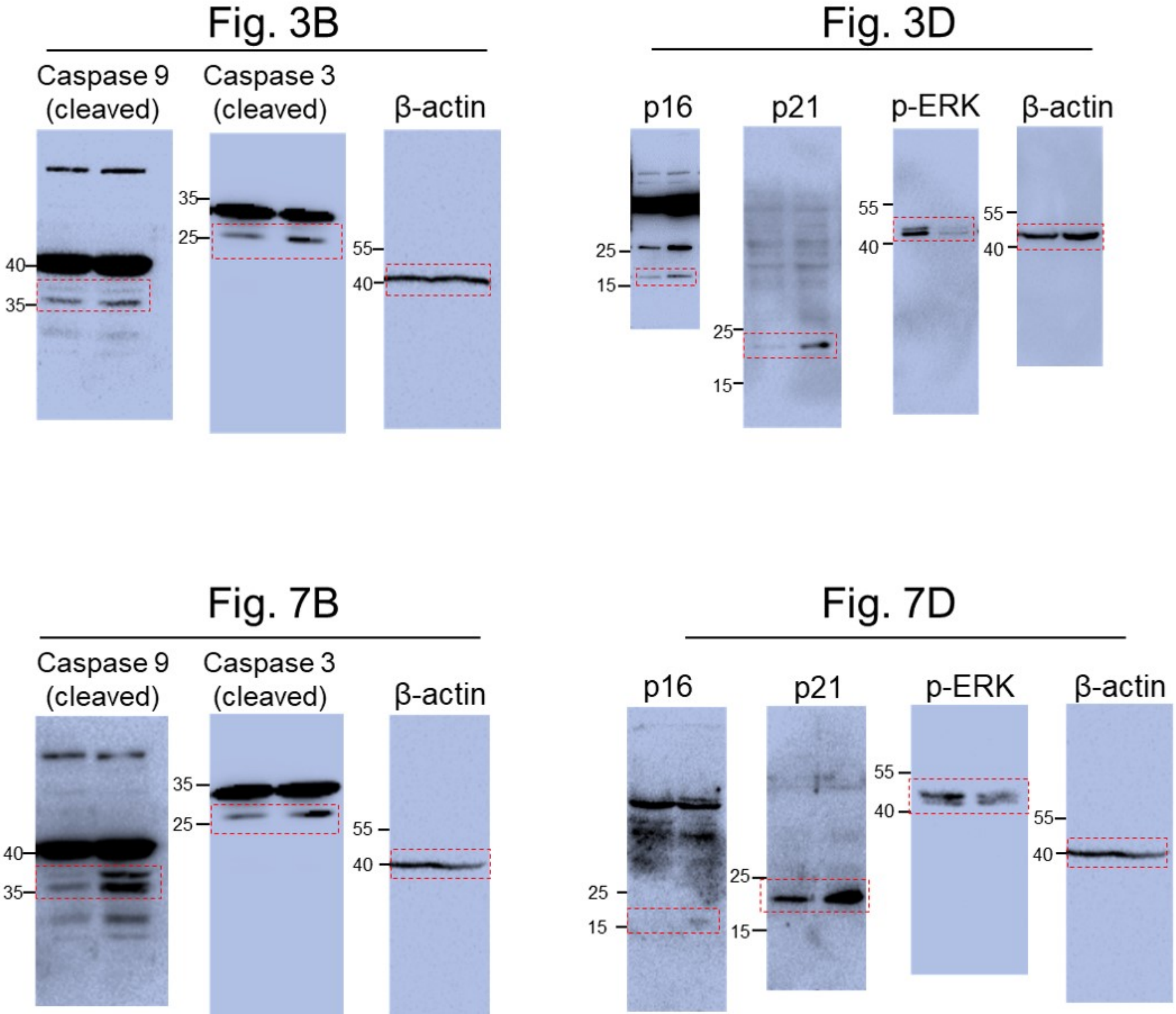

Supplement: Supplementary file 1 — Supplementary figures. [file jcav14p0050s1.pdf]
